# Editorial: Methods and applications of CRISPR technology in plant sciences, 2022

**DOI:** 10.3389/fpls.2023.1207257

**Published:** 2023-05-04

**Authors:** Ljudmilla Timofejeva, Davinder Singh

**Affiliations:** ^1^ Department of Plant Biotechnology, Centre of Estonian Rural Research and Knowledge, Jõgeva, Estonia; ^2^ School of Life and Environmental Sciences, Faculty of Science, The University of Sydney, Sydney, NSW, Australia

**Keywords:** CRISPR, Cas nucleases, DNA-free transformation, hairy root, gene editing

The Clustered Regularly Interspaced Short Palindromic Repeats (CRISPR)/Cas-mediated gene editing is a rapidly developing technique to investigate fundamental questions in Plant Science research and genetic improvement of plants. This system has been shown to work in over 70 different crop species from apple to wheat. Generation of site-specific mutations in genes of interest requires expression of Cas (CRISPR-associated) nuclease and guide RNA (gRNA) in plant cells. Efficiency of the CRISPR/Cas approach depends on several factors including but not limited to: (i) the type of Cas nuclease optimized to plant codons (ii) the presence and number of the NLS that can affect the Cas9 delivery to the cell nuclei, (iii) source and nature of promoters used to drive the expression of Cas genes, and (iv) an appropriate choice of target site and construction of gRNAs which can increase the mutation frequency. Finally, the editing success is contingent on the type of transgene delivery to the host/plant genome.

The purpose of this Research Topic was to provide a platform for the publication of well-written protocols for creating novel CRISPR vectors, and various plant transformation methods available for a wide range of plant species. Six papers were published including two - original research, one – methods, and three - reviews.

In the first review paper, Son and Park provided in-depth review on four key issues relevant to plant genome editing: (i) plant organelle genome editing; (ii) transgene-free genome editing; (iii) virus-induced genome editing; and (iv) editing of recalcitrant elite crop inbred lines. This review provides an up-to-date and comprehensive summary on the state of CRISPR/Cas9-mediated genome editing in plants that will push this technique forward. In addition to the opportunities, the review also addressed certain challenges and issues on future utilization of CRISPR/Cas9 technology in crop improvement. This paper also provides very well-defined figures especially on schematic overview of strategies for the generation and isolation of transgene-free edited plants and schematic illustration of tobacco rattle virus (TRV)-mediated seed genome editing in tobacco relatives.

Review article by Sustek-Sanchez et al. specifically covers improvement of abiotic stress tolerance of forage grasses using genome editing. The authors provided an overview of the main metabolic and molecular changes that plants suffer in order to cope with the effects of abiotic stress derived from climate change adversities. The review proposes how the new genetic resources and genome editing tools can be used to improve forage grass breeding that will help achieve food security in a sustainable way. Four abiotic stresses (heat, low temperature, drought, salinity) hinder the overall wellbeing of a non-tolerant grasses and using the CRISPR-Cas system, different genes can be targeted that can lead towards the generation of abiotic stress tolerant plants. The review also touches on the latest developments in the regulatory framework for genome editing, especially with regard to the EU, and identify factors affecting the application of genome editing techniques for the improvement of grasses.

The third review by Khan et al. discusses the prospects, applications and limitations of CRISPR/Cas based gene editing for genetic improvement of commercial palms (date palm, coconut palm, and oil palm). It reviews the options available for GE technologies, the application of proposed methods for genetic transformation, and regulatory pathways for genetic improvement in the palm family. The authors also address the bioengineering role of fatty acid biosynthesis in enhancing the commercial production of vegetable oil for human consumption. We find this review highly illustrative with seven well-crafted figures which can be highly valuable for teaching and demonstrative presentations.

Cauliflower mosaic virus (*CaMV*) *35S* promoter is the widely used promoter to drive Cas9 expression. The original research article by Jedličková et al. in this topic compared the efficiency of long (1.3 kb) and short (0.4 kb) versions of the *35S* promoter in four constructs for each version and found that the longer promoter was slightly more efficient than its short version. In addition, the authors tested the effect of the Arabidopsis *RbcS2B* promoter driving *Staphylococcus aureus* Cas9 expression. The authors analysed the ability to generate mutations, their variety and frequency in targeted loci/sequences of *B. napus BnaTAA1* gene by two Cas9 nucleases: from *Staphylococcus aureus* and plant-codon optimized *Streptococcus pyogenes.* It was further demonstrated that Cas9 from *Streptococcus pyogenes* is more effective. It is known that a SV40 nuclear localization signal (NLS) sequence fused to the Cas9 gene can also improve the efficiency of generating mutations as it delivers Cas9 to the genomic nuclei. The effect of the N-terminal NLS was tested in five constructs and allowed the authors to demonstrate a 25% increase in mutagenesis due to the presence of NLS.

Considering not whole plant but its part transformation, it is important to use a strong promoter active in the tissue to be transformed. A constitutive promoter driving Cas9 expression is not able to produce homozygous mutants required for loss-of function-studies with a high frequency. Of special interest in this issue is the methods paper by Liu et al. who identified a highly efficient *Arabidopsis thaliana* gamma-*glutamilcysteine synthetase* promoter (*AtGCSpro*) to drive Cas9 expression in root meristem and in the whole developing roots. Preliminary, the *AtGCSpro* high activity in driving GUSPlus expression was confirmed in many eudicot species and then activity of promoter with different lengths of promoter region upstream of the translation start site of gamma-*glutamylcysteine synthetase* gene was tested in soybean hairy roots. Authors observed the lower activity of *AtGCSpro*_1178_ and no activity of *AtGCSpro*_833_ in driving GUS expression. In addition, activity of *AtGCSpro* was compared with the *Ubiquitin* promoter and *YAO* promoter (a germ cell-specific promoter with high activity in roots). Authors conclude that *AtGCSpro* is the most efficient promoter for inducing homozygous or biallelic mutations, outperforming the *Ubiquitin, YAO* and *CaMV 35S* promoters in hairy roots.

In their research paper, Subburaj et al. described the system based on the direct delivery of Cas9 ribonucleoprotein (RNP) complex to soybean protoplasts isolated from young seedlings. PEG-mediated transfection of protoplasts with preassembled Cas9 RNP complex is fast and low-cost approach for developing mutant lines for plant biology and biotechnology studies. A DNA-free delivery system dramatically reduces the effects related to the undesirable introduction of vector DNA and random integration of recombinant DNA into the plant genome. To empirically test their platform, the authors selected a model gene from the soybean genetic toolbox and used five different guide RNA (gRNA) sequences that targeted the *constitutive pathogen response 5* (*CPR5*) gene associated with the growth of trichomes in soybean. Based on targeted mutagenesis insertion and deletion frequency and sequences, the authors were able to identify different mutation patterns within insertions and deletions (InDels). The authors demonstrated that DNA-free delivery of Cas9 complexes to protoplasts is a useful approach to perform early-stage genetic screens and analysis of Cas9 activity in soybeans.

We are confident that readers will find all six articles in this Research Topic very useful for research, teaching, and application of newly and rapidly emerging CRISPR/Cas9 technology and assays.

## Author contributions

All authors listed have made a substantial, direct, and intellectual contribution to the work and approved it for publication.

